# Implications of constructed biologic subtype and its relationship to locoregional recurrence following mastectomy

**DOI:** 10.1186/bcr3197

**Published:** 2012-05-23

**Authors:** Laura S Dominici, Elizabeth A Mittendorf, Xumei Wang, Jun Liu, Henry M Kuerer, Kelly K Hunt, Abenaa Brewster, Gildy V Babiera, Thomas A Buchholz, Funda Meric-Bernstam, Isabelle Bedrosian

**Affiliations:** 1 Department of Surgical Oncology, University of Texas MD Anderson Cancer Center, 1515 Holcombe Blvd, Houston, TX 77030, USA; 2 Department of Breast Medical Oncology, University of Texas MD Anderson Cancer Center, 1515 Holcombe Blvd, Houston, TX 77030, USA; 3 Department of Biostatistics, University of Texas MD Anderson Cancer Center, 1515 Holcombe Blvd, Houston, TX 77030, USA; 4 Department of Radiation Oncology, University of Texas MD Anderson Cancer Center, 1515 Holcombe Blvd, Houston, TX 77030, USA

## Abstract

**Introduction:**

We examined the prognostic value of biologic subtype on locoregional recurrence (LRR) after mastectomy in a cohort of low risk women who did not receive adjuvant radiation therapy.

**Methods:**

A total of 819 patients with invasive breast cancer underwent mastectomy from January 2000 through December 2005. No patient received preoperative chemotherapy. Estrogen receptor (ER) receptor, progesterone receptor (PR) and human epidermal growth factor receptor 2 (HER2) status were used to construct the following 4 subtypes: i) ER+ or PR+ and HER2- (HR+/HER2-), ii) ER+ or PR+ and HER2+ (HR+/HER2+), iii) ER- and PR- and HER2+ (HR-/HER2+)and iv) ER- and PR- and HER2- (HR-/HER2-). LRR-free survival was estimated by the Kaplan-Meier method. Cox proportional hazard models were used to evaluate the association between time-to-event outcomes and patient prognostic factors.

**Results:**

At a median follow-up of 58 months, five-year cumulative incidence of LRR for the entire cohort was 2.5%. Subtype specific LRR rates were 1% for HR+/HER2-, 6.5% in HR+/HER2+, 2% for HR-/HER2+ and 10.9% for HR-/HER2- (*P *< 0.01). In HER-2+ patients (irrespective of ER/PR status), trastuzumab therapy was not associated with LRR-free survival. On multivariate analysis, one to three positive lymph nodes (HR 4.75 (confidence interval (CI) 1.75 to 12.88, *P *< 0.01), ≥ 4 positive lymph nodes (HR23.4 (CI 4.64 to 117.94, *P *< 0.01), HR+/HER2+ (HR 4.26 (CI 1.05 to 17.33), *P *= 0.04), and HR-/HER2- phenotype (HR 13.87 (CI 4.96 to 38.80), *P *< 0.01) were associated with shorter LRR-free survival whereas age > 50 at diagnosis (HR 0.31 (CI 0.12 to 0.80), *P *= 0.02) was associated with improved LRR-free survival. Among the HR-/HER2- subtypes, five-year LRR incidence was 23.4% in patients with positive lymph nodes compared to 7.8% for lymph node negative patients (*P *= 0.01), although this association did not reach significance when the analysis was limited to HR-/HER2- women with only one to three positive lymph nodes (15.6% versus 7.8%, *P *= 0.11).

**Conclusions:**

Constructed subtype is a prognostic factor for LRR after mastectomy among low risk women not receiving adjuvant radiation therapy, although rates of LRR remain low across subtypes. Patients with node positive, HR-/HER2- type tumors were more likely to experience LRR following mastectomy alone. Prospective studies to further investigate the potential benefit of adjuvant radiation therapy in these women are warranted.

## Introduction

The identification of biologic subtypes of breast cancer has provided an important window into the underlying heterogeneity of this cancer and has provided a new paradigm for classification of this disease [1]. Since the initial demonstration that these molecular subtypes correlated with differences in survival [2], other groups have similarly confirmed the clinical relevance of the biologic subtypes [3,4]. HER-2 amplified and basal subtypes are associated with significantly worse overall survival while the luminal A subtype has a higher expression of genes related to the estrogen receptor and is associated with the best prognosis. Although the initial description of molecular profiles of breast cancer was based on transcriptional profiling, the practical challenges of this approach have led many to use clinical approximations of these subtypes primarily using ER, PR and HER2. Importantly, much of the clinical data on patient related outcomes based on subtype utilizes this clinical approximation, underscoring that they yield results similar to those of studies utilizing the full gene profile [5-7].

The impact of biologic subtypes on local regional recurrence (LRR) outcomes has been less well studied. Recent data in women with early stage breast cancer undergoing breast conserving therapy suggests that these biologic subtypes also confer a similar risk profile for local recurrence as they do for distant disease [7-9]. Among women undergoing mastectomy, similar trends appear to hold [9,10].

The role of chest wall and regional nodal irradiation following mastectomy continues to evolve [11]. In many cases, women undergoing mastectomy will not require radiation therapy. However, based on clinical features, some women felt to be at high risk for recurrence following mastectomy will be recommended for post-mastectomy radiation therapy (PMRT). Biologic subtype of breast cancer is not currently considered in the indications for PMRT, as increased risk for LRR based on subtype has not been as well established. The objectives of this study were to determine the relevance of constructed biologic subtype (using ER, PR and HER2 as surrogates) in predicting LRR following mastectomy in a cohort of women deemed clinically low risk for locoregional recurrence who, thus, did not undergo PMRT.

## Materials and methods

### Patient selection

The study cohort included 819 women with invasive breast cancer who underwent mastectomy at the University of Texas MD Anderson Cancer Center from January 2000 through December 2005. Seven patients who presented with breast cancer were found to have a contralateral cancer on imaging at presentation or on prophylactic mastectomy pathology. In these patients, the index breast cancer was used for analysis. Patients with recurrent ipsilateral breast cancer after previous breast conservation therapy were excluded. Two patients with Stage IV disease were included in this analysis. Neither received PMRT and both were found to be Stage IV on postoperative staging work-up. Patients receiving preoperative systemic therapy were excluded from this analysis. No patient in this study received adjuvant radiation therapy.

This study was approved by the MD Anderson Cancer Center Institutional Review Board and waiver of consent provided by the IRB; specific consent from the patients to participate in the study and consent to publish the resulting data was not required by the IRB.

### Margin assessment

Margins were deemed positive only if there was tumor on ink. Close margins were defined as tumor within 2 mm of ink. DCIS or invasive cancer > 2 mm from ink was classified as a negative margin.

### Treatment

Adjuvant systemic therapy was administered at the discretion of the treating oncologist. Although adjuvant trastuzumab was not routinely used during the time of this study, 28 out of 108 HER2 + patients in our cohort were additionally treated with adjuvant trastuzumab. Women with hormone receptor positive tumors (ER and or PR > 10%) were advised to receive adjuvant hormonal therapy. Twelve patients with ER negative (< 10%) and PR negative (< 10%) cancers received hormonal therapy, most for weakly hormone receptor positive tumors (1% to 9% positive).

### Follow up

Patients were generally seen in follow-up every three months for the first two years and, subsequently, every six months. Patients who did not routinely follow-up at MD Anderson Cancer Center were contacted by phone or mail for follow-up information. Follow-up time was counted from date of diagnosis to the date of death, date of first event or last confirmed date of breast cancer disease-free status. The median follow up time was 58 months (range 2.4 to 122 months).

### Classification of biologic subtype

Four subtypes were constructed based on the receptor status of the primary tumor: i) ER+ or PR+ and HER2- (HR+/HER2-), ii) ER+ or PR+ and HER2+ (HR+/HER2+), iii) ER- and PR- and HER2+ (HR-/HER2+)and iv) ER- and PR- and HER2- (HR-/HER2-). ER and PR were considered positive if immunohistochemistry (IHC) staining was 10% or greater [12]. An IHC score of 3+ or HER2 amplification by fluorescence *in situ *hybridization (FISH) score was used to determine HER2 positive status. In the setting of 2+ IHC staining and no FISH data, tumors were considered negative for HER2 [13]. Patients with unknown biologic subtype constituted those where HER2 testing was not performed. These patients were included when characterizing the patient population as a whole, but were excluded from subtype specific analyses.

### Statistical analysis

The primary end point of this study was time to local or regional recurrence, defined as biopsy-proven recurrence in the chest wall, skin, axilla, infraclavicular nodes, internal mammary nodes or supraclavicular nodes. The local or regional recurrence free time was defined as the time interval from the surgery date to the local or regional recurrence date, death date or last follow-up date, whichever occurred first.

Pearson's Chi-Square tests were used to assess the association of clinical variables among subtypes. The Kaplan-Meier method [14] was used to estimate the probability of overall survival as well as local or regional recurrence-free survival. The long-rank test [15] was used to compare the time-to-event outcomes among subgroups of patients. The Cox proportional hazards models [16] were used to assess the association between the time-to-event outcomes and patient prognostic factors as well as constructed subtypes. Backward model selection methods were used to determine the final fitted models. *P*-values less than 0.05 were deemed statistically significant. All statistical analyses were conducted in SAS 9.1 for Windows (SAS Institute Inc., Cary, NC).

## Results

The characteristics of the 819 women in this analysis are described in Table 1. The majority of patients presented with early stage, node negative breast cancer. In this mostly post-menopausal cohort, 80% had hormone sensitive tumors as measured by ER or PR staining. Thirteen percent of the women had HER2 overexpressing tumors; of these, only one quarter received trastuzumab therapy.

**Table 1:** Patient baseline characteristics (number = 819).

Characteristic	Number	%
Age at diagnosis		
< 50	290	35.4
> 50	529	64.6
T stage		
T1	613	74.8
T2	195	23.8
T3	11	1.4
Number of positive nodes		
0	586	71.6
1-3	219	26.7
> 4	10	1.2
No nodes sampled	4	0.5
Grade		
1	84	10.3
2	431	52.6
3	300	36.6
Unknown	4	0.5
ER or PR positive	656	80.1
HER2 positive	109	13.3
LVI present	137	16.7
Hormonal therapy received	553	67.5
Chemotherapy received	389	47.5
Trastuzumab therapy received (if HER2 positive, Number = 108)	28	25.9
Margins		
Close or positive	27	3.3
Negative	791	96.6
Unknown	1	0.1
Constructed subtype		
HR+/HER2+	574	70
HR+HER2+	57	7
HR-/HER2+	51	6.2
HR-/HER2-	94	11.5
Unknown	43	5.3

ER, estrogen receptor; HER2, human epidermal growth receptor 2; LVI, lymphovascular space invasion; PR, progesterone receptor.

There were significant differences across the constructed subtypes with regard to clinical, pathological and treatment variables (Table 2), including median age at presentation (*P *< 0.01), number of positive nodes (*P *= 0.02) and grade (*P *< 0.01). Women with HR-/HER2+ tumors tended to be younger, while those with HR-/HER2- tumors has the lowest incidence of nodal involvement. Compared to all other cohorts, women with HR+/HER2- had the lowest probability of grade 3 tumors. As expected, the number of patients receiving hormonal therapy and chemotherapy was also significantly different (all *P *< 0.01) with the majority of women with ER and/or PR positive tumors receiving endocrine therapy and the majority of patients with HER2+ (irrespective of ER/PR status) and HR-/HER2- tumors receiving chemotherapy.

**Table 2:** Patient characteristics by breast cancer subtype (Number = 819).

Variable	All patients (Number = 819)	HR+/HER2- (Number = 574)(%)	HR+/HER2+ (Number = 57)(%)	HR-/HER2+ (Number = 51)(%)	HR-/HER2- (Number = 94)(%)	*P*-value
Median age at diagnosis	55	56	54	49	54	< 0.01
T1	615	439 (76.48)	41 (71.93)	36 (70.59)	60 (63.83)	0.06
Positive lymph nodes	230	164 (28.57)	25 (43.86)	14 (27.45)	19 (20.21)	0.02
≥ 4 positive lymph nodes	10	7 (1.22)	0	0	3 (3.19)	0.25
Lymphovascular invasion	137	89 (15.51)	16 (28.07)	13 (25.49)	16 (17.02)	0.07
Modified Black's nuclear grade 3	301	132 (23.00)	36 (63.16)	48 (94.12)	75 (79.79)	< 0.01
Close or positive margins	27	17 (2.96)	0	3 (5.88%)	5 (5.32)	0.21
Hormonal therapy	556	473 (82.40)	50 (87.72)	5 (9.80)	6 (6.38)	< 0.01
Chemotherapy	390	235 (40.94)	42 (73.68)	38 (74.51)	64 (68.09)	< 0.01
Median follow-up (months)	58	58	55	62	53	0.11
Locoregional recurrence incidence rate at 60 months (95%CI)	2.5% (1.6% to 4.0%)	1% (0.1% to 1.9%)	6.5% (0 to 13.5%)	2% (0 to 5.8%)	10.9% (3.8% to 17.5%)	< 0.01
Local recurrence incidence rate at 60 months (95% CI)	0.9% (0.5% to 2.1%)	0.5% (0.2% to 1.7%)	4.7% (2.3% to 17.7%)	0%	2.6% (0.7% to 10.5%)	0.06
Regional recurrence incidence rate at 60 months (95% CI)	1.5% (0.9% to 2.8%)	0.5% (0.2% to 2.0%)	1.8% (0.3% to 12.1%)	2.0% (0.3% to13.4%)	8.3% (4.2% to 16.1%)	< 0.01

CI, confidence interval; HER2, human epidermal growth receptor 2; HR, hormone receptor.

**Figure 1:**
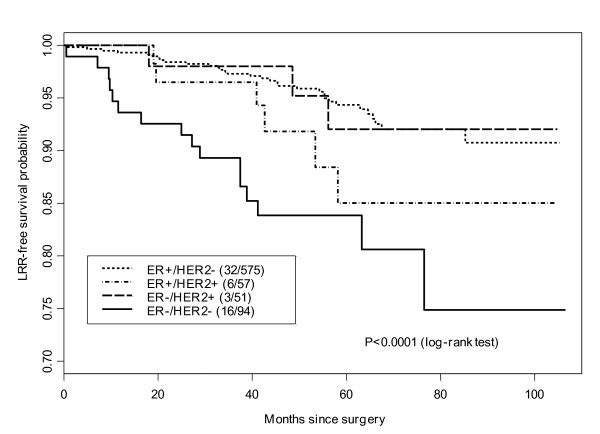
**Log rank test of local regional recurrence free survival by tumor subtype**.

**Figure 2:**
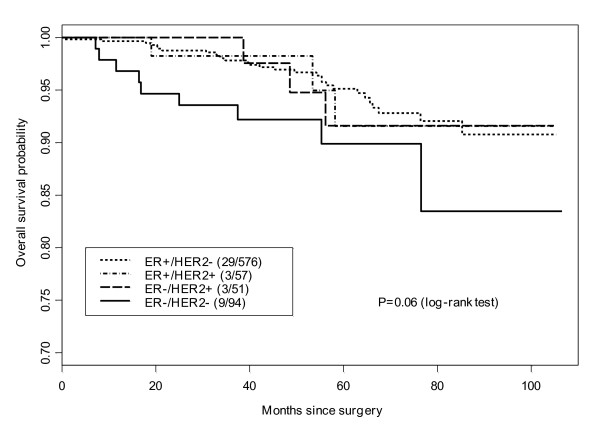
**Log rank test of overall survival by tumor subtype**.

With a median follow-up of 58 months, the five-year cumulative incidence of LRR was 2.5%. For patients with HR+/HER2-, the five-year incidence of LRR was 1% (95%CI, 0.1% to 1.9%), compared with 6.5% (95%CI, 0 to 13.5%) in the HR+/HER2+ group, 2% (95% CI, 0 to 5.8%) for HR-/HER2+ and 10.9% (95% CI, 3.8 to 7.5%) in the HR-/HER2- group (*P *< 0.01) (Table 2 and Figure 1). Although there were differences in overall survival among the different subgroups, these did not reach statistical significance (*P *= 0.06) (Figure 2).

On univariate analysis, younger age at presentation was associated with greater risk of LRR (HR 3.22 (95%CI 1.28 to 8.33), *P *= 0.01) whereas ER- or PR-positive tumors were associated with markedly lower risk of LRR (HR 0.15 (95%CI 0.06 to 0.38), *P *< 0.01) (Table 3). Significant predictors of locoregional failure also included increasing tumor size, nodal involvement, increasing pathologic stage, high grade and presence of lymphovascular space invasion (LVI) (all *P *< 0.05). Among the subtypes, HR-/HER2- had the highest risk of LRR (HR13.6 (95% CI 4.63 to 39.7) *P *< 0.01). HER2 status, when analyzed independent of HR status, was not significantly associated with LRR (HR 1.64, 95%CI 0.54 to 4.94, *P *= 0.38). However, within the constructed subtypes women with HR+/HER2+ disease, but not those with HR-/HER2+ disease, had significantly increased risk of LRR when compared to HR+/HER2- tumors (HR 6.25 (95%CI 1.49 to 6.2), *P *= 0.01 and HR 2.24 (95%CI 0.26 to 19.2), *P *= 0.46, respectively). Administration of trastuzumab to HER2+ patients did not significantly alter LRR risk (HR 3.87 (95%CI 0.53 o 28.1), *P *= 0.18).

**Table 3:** Univariate Cox PH regression models for local/regional recurrence(LRR)-free survival (Number = 819).

Variable	LRR-free (Number = 800)	LRR (Number = 19)	HR (95%CI)	*P*-value
Age at diagnosis				
> 50	522	7	reference	
< 50	278	12	3.22 (1.28 to 8.33)	0.01
T stage				
T1	604	9	reference	
T2	187	8	2.95 (1.14-7.64)	0.03
T3	9	2	19.8 (4.24-92.3)	< 0.01
Pathologic stage				
I	450	4	Reference	
IIA and IIB	335	11	3.74 (1.19-11.7)	0.02
IIIA, IIIB, IIIC and IV	15	4	31.8 (7.94- 127)	< 0.01
Number of positive lymph nodes				
0	579	7	reference	
1-3	209	10	3.94 (1.50-10.3)	0.01
> 4	8	2	23.3 (4.83- 112)	< 0.01
Modified Black's nuclear grade				
3 versus 1,2			6.93 (2.30-20.88)	< 0.01
ER or PR				
Negative	137	11	reference	
Positive	648	8	0.15 (0.06-0.38)	< 0.01
HER2+				
Negative	653	15	reference	
Positive	105	4	1.64 (0.54-4.94)	0.38
Lymphovascular invasion				
Negative	555	9	reference	
Positive	127	10	5.08 (2.06-12.5)	< 0.01
Received trastuzumab (in HER2+ patients)				
No	78	2	reference	
Yes	26	2	3.87 (0.53-28.1)	0.18
Systemic therapy				
No	141	3	reference	
Yes	658	16	1.11 (0.32-3.80)	0.87
Constructed Subtype				
HR+/HER2-	569	5	reference	
HR+/HER2+	54	3	6.25 (1.49-26.2)	0.01
HR-/HER2+	50	1	2.24 (0.26-19.2)	0.46
HR-/HER2-	84	10	13.6 (4.63-39.7)	< 0.01

CI, confidence interval; ER, estrogen receptor; HER2, human epidermal growth receptor 2; PR, progesterone receptor.

On multivariate analysis, ≥ 4 positive nodes and HR-/HER2- subtype were the strongest predictors of LRR failure (HR 23.4 (95%CI 4.64 to 117.94) and HR 13.87 (95%CI 4.96 to 38.8), respectively, *P *< 0.05) (Table 4). Other variables that retained significant association with LRR risk were one to three positive nodes (HR 4.75 (95%CI 1.75 to 2.88), *P *= 0.02), age at diagnosis < 50 (HR 3.23 (95%CI 1.25 to 8.33), *P *= 0.02) and HR+/HER2+ subtype (HR 4.26 (95%CI 1.05 to 7.33), *P *= 0.04).

**Table 4:** Multivariable Cox PH regression models for local/regional recurrence-free survival (Number = 819).

Variable	HR (95%CI)	*P*-value
1-3 positive lymph nodes	4.75 (1.75 to 12.88)	< 0.01
≥ 4 positive lymph nodes	23.40 (4.64 to 117.94)	< 0.01
Age < 50 at diagnosis	3.23(1.25 to 8.33)	0.02
HR+/HER2+ Subtype	4.26 (1.05 to 7.33)	0.04
HR-/HER2- Subtype	13.87 (4.96 to 38.80)	< 0.01

CI, confidence interval; HER2, human epidermal growth receptor 2; HR, hormone receptor.

We next explored whether women with HR-/HER2- tumors could be further stratified for LRR risk by clinical variables. The presence of lymphovascular space invasion was also associated with increased risk of LRR within the HR-/HER2- subtype (29.7% versus 9.23%, HR = 3.44, *P *= 0.04) (Table 5). Similarly, patients with node positive disease had increased probability of LRR at five years compared to those who did not (23.4% versus 7.8%, HR = 4.44, *P *= 0.01) (Table 6). When women with ≥ 4 nodes were removed from this analysis, LRR among those women with one to three positive nodes remained elevated but did not reach statistical significance (15.6% versus 7.8%, *P *= 0.11).

**Table 5:** Locoregional recurrence within HR-/HER2- subtype by presence of lymphovascular invasion (LVI) (Number = 80)

Variable	Locoregional recurrence	No recurrence	LRR probability at 60 month	*P*-value
LVI positive	4	12	29.7%	
LVI negative	6	58	9.2%	0.04

**Table 6:** Locoregional recurrence within HR-/HER2- subtype by lymph node status (Number = 94).

Variable	Locoregional recurrence	No recurrence	LRR probability at 60 month	*P*-value
Node positive	5	14	23.4%	
Node negative	5	70	7.8%	0.01

HER2, HER2, human epidermal growth receptor 2; HR, hormone receptor; LRR, locoregional recurrence.

## Discussion

Classification of breast cancer by biologic subtype has proven to be a strong predictor of distant relapse [2,3]. Increasingly, data are emerging to support the role of tumor biology as a determinant of local and regional failure. In this report, we show that risk of chest wall and regional nodal failure following mastectomy among a cohort of women who did not receive adjuvant radiation therapy is significantly different based on tumor subtype. Specifically, while the overall risk of loco-regional failure is low in this cohort, it rises modestly in women with HR+/HER2+ and significantly in women with HR-/HER2- tumors. Even among women with HR-/HER2- tumors, it is possible to further stratify between those at modest risk and those at marked risk of LRR by the presence of LVI and nodal involvement.

Although direct comparisons with other series is difficult given differences between cohorts, the pattern of relatively higher risk among HR-/HER2- subtypes and lower risk among HR+/HER2- subtypes seen in our patient population is consistent with other series reporting on LRR following mastectomy. In a series of 1,000 high risk women, Kyndi *et al. *reported five-year rates of LRR of 20% among women with HR-/HER2- tumors who received PMRT compared to 3% in the subset of patients with HR+/HER2- tumors receiving PMRT [10]. Using a six biomarker panel, Voduc *et al. *reported ten-year LRR of 19% in tumors negative for ER, PR and HER2 but expressing either CK5/6 or EGFR and 13% among those tumors negative for all biomarkers [9]. In contrast, ten-year LRR in women with HR+/HER2- and low ki-67 tumors was 8%. This rate of locoregional failure appears high in this subgroup of women with estrogen responsive tumors, which is likely explained by a sizeable number of women in this study who did not receive adjuvant endocrine therapy despite expression of hormone receptors. Thus, the actual rate of LRR following mastectomy in women with HR+/HER2- tumors is likely closer to that reported by our series and the series by Kyndi *et al.*

We also did not find adjuvant trastuzumab to impact LRR significantly. Results from both NSABP B-31 and NCCTG N9831 trials demonstrate an approximately 50% reduction in combined local and regional events in the arm randomized to receive adjuvant trastuzumab [13]. Therefore, it is very likely that the lack of effect we noted in our series is due to the small number of the HER2+ patients who received trastuzumab.

An important difference between our studies and those previously reported is the unexpectedly low rate of LRR seen in our series among women with HR-/HER2+ tumors (2%). This stands in distinction with the higher rate of LRR seen among those with HER2+ tumors who also express hormone receptors (HR+/HER2+) within our own series (6.5% versus 2%). While only 25% of the total HER2+ cohort received trastuzumab, there were no differences in the proportion of women who received this treatment based on ER status (28% in the HR+/HER2+ group versus 24% in the HR-/HER2+ group). Thus, the differences we report in LRR rates between the two HER2+ subsets may be due to either small sample size or, potentially, differences in responsiveness to trastuzumab-based therapy. Larger datasets are required to further investigate this possibility.

Lastly, our data demonstrate that while tumor subtypes are associated with LRR outcomes, further stratification by clinical variables classically associated with increased risk remains valuable. Thus, women with HR-/HER2- tumors, who have either positive nodes or LVI, are at markedly increased risk of LRR. This is similar to the study by Abdulkarim *et al. *who reported that LVI and nodal involvement were independently associated with LRR in women with HR-/HER2- breast cancer [17]. These authors also noted that women with early stage, node negative, HR-/HER2- breast cancer treated with breast conserving therapy (BCT) had lower five-year LRR risk compared to a similar cohort treated with mastectomy without radiation. Rates of distant disease were not significantly different. Nonetheless, the authors suggest that the benefit of adjuvant radiation therapy needs to be investigated in all women with HR-/HER2- breast cancer. However, our data show five- year LRR risk for women who are node negative to be well under 10%, the threshold generally used for administration of adjuvant radiation. Thus, the majority of such women, if given radiation, would be expected to derive no benefit. In contrast, for women with HR-/HER2- tumors and one to three positive nodes, we found a trend towards increase risk of LRR (5.6% versus 7.8%, *P *= 0.11). While the difference in our study did not reach statistical significance, this is likely due to small sample size and adjuvant radiation therapy in this subset of HR-/HER2- breast cancers may be warranted to improve both local-regional control and, based on meta-analysis of randomized clinical trials by the Early Breast Cancer Trialists' Collaborative Group [18], survival as well.

## Conclusions

In summary, we found that constructed subtypes can differentiate risk of LRR following mastectomy among a cohort of women generally considered at low risk for LRR. Overall, the risk of LRR in our study remains quite low across subtype. The addition of clinical risk variables to HR-/HER2- subtype can identify a subset of women at marked increase for LRR and for whom adjuvant radiation may be considered.

## Abbreviations

CI: confidence interval; CK: cytokeratin; DCIS: ductal carcinoma *in situ*; EGFR: epidermal growth factor receptor; ER: estrogen receptor; FISH: fluorescence *in situ *hybridization; HR: hormone receptor; HER2: human epidermal growth receptor 2; IHC: immunohistochemistry; LRR: locoregional recurrence; LVI: lymphovascular space invasion; NSABP: National Surgical Adjuvant Breast and Bowel project; NCCTG: North Central Cooperative Trials Group; PMRT: post-mastectomy radiation therapy; PH: proportional hazards; PR: progesterone receptor.

## Competing interests

The authors declare that they have no competing interests.

## Authors' contributions

LD participated in data collection and preparation of the manuscript. XM and JL led the data analysis and participated in manuscript preparation. AB participated in data analysis. EAM, HMK, KKH, GVB, TAB and FMB all participated in data interpretation and critical revision of the manuscript. IB conceived of the study, participated in data collection and analysis and drafting of the manuscript. All authors have read and approved the final manuscript for publication.
